# Candelilla Wax Extracted by Traditional Method and an Ecofriendly Process: Assessment of Its Chemical, Structural and Thermal Properties

**DOI:** 10.3390/molecules27123735

**Published:** 2022-06-10

**Authors:** Itzel C. Núñez-García, Linda G. Rodríguez-Flores, Michelle H. Guadiana-De-Dios, María D. González-Hernández, Guillermo C. G. Martínez-Ávila, José A. Gallegos-Infante, Rubén González-Laredo, Walfred Rosas-Flores, Victor J. Martínez-Gómez, Romeo Rojas, Ignacio Villanueva-Fierro, Miriam Rutiaga-Quiñones

**Affiliations:** 1 Departamento de Ings. Química-Bioquímica, TecNM/Instituto Tecnológico de Durango, Blvd. Felipe Pescador 1830 Ote., Nueva Vizcaya, Durango 34080, Durango, Mexico; itzelng8@gmail.com (I.C.N.-G.); 17040845@itdurango.edu.mx (L.G.R.-F.); agallegos@itdurango.edu.mx (J.A.G.-I.); rubenfgl@itdurango.edu.mx (R.G.-L.); wrosas@itdurango.edu.mx (W.R.-F.); v.martinez@itdurango.edu.mx (V.J.M.-G.); 2 Laboratorio de Química y Bioquímica, Facultad de Agronomía, Universidad Autónoma de Nuevo León, General Francisco Villa S/N, Ex-Hacienda “El Canadá”, General Escobedo 66050, Nuevo León, Mexico; michelle.guadianade@uanl.edu.mx (M.H.G.-D.-D.); lolis.90.6@gmail.com (M.D.G.-H.); romeo.rojasmln@uanl.edu.mx (R.R.); 3 Cátedras-Conacyt TecNM/Instituto Tecnológico de Durango, Blvd. Felipe Pescador 1830 Ote., Nueva Vizcaya, Durango 34080, Durango, Mexico; 4 Instituto Politécnico Nacional, CIIDIR-Unidad Durango, Calle Sigma 119, 20 de Noviembre II, Durango 34220, Durango, Mexico; ivillanuevaf@ipn.mx

**Keywords:** candelilla wax, eco-friendly process, traditional process, properties

## Abstract

A comparative study was carried out on the chemical, structural and thermal properties of candelilla wax from four wax-producing communities in Mexico, which was obtained by two extraction processes, the conventional one using sulfuric acid (SA) and an eco-friendly alternative process using citric acid (CA) as the extracting agent. The waxes were analyzed by basic chemistry (acidity, saponification, ester indexes, and others), color, Fourier transform infrared spectroscopy (FTIR), Raman micro-spectroscopy, X-ray diffraction (XRD), differential scanning calorimetry (DSC), and hardness and brittleness measurements. The waxes obtained by the environmentally friendly process showed differences in their physicochemical properties when compared to waxes from the conventional process. In addition, they showed some improvements, such as lighter shades and harder waxes, suggesting that the new environmentally friendly process is a viable option.

## 1. Introduction

Candelilla plant (*Euphorbia antisyphilitica* Zucc) is endemic to the arid and semiarid regions of Northern Mexico, and it grows into clusters of nearly leafless and thin stems covered in wax that protects the stem as thick layers, giving tolerance against abiotic (i.e., cold and hot temperatures) and biotic (i.e., insect attacks) agents [1]. This plant is the source used for the extraction of Candelilla wax (CW), which has many applications in the food industry, as it can be used in the development of biodegradable edible coatings and films, providing several advantages to food products such as protection from microbial spoilage, enhancement of product quality, and an extension of shelf-life, among other functional properties [1,2,3,4,5,6,7]. In addition, CW can be applied in the formulation of oleogels, which have arisen as a promising alternative for maximizing its health benefits that have been known in diverse food, cosmetic, and pharmaceutical products for more than hundred years [8,9,10,11,12].

In the north of Mexico, mainly in Coahuila, Durango and Zacatecas, the CW extraction is a relevant economic activity for marginalized and rural communities of these states. The traditional process used involves the use of hot sulfuric acidic; however, this extraction method represents several technical, environmental, and health disadvantages due to the use of this strong acid. Therefore, important changes and regulations must be done to apply more green techniques in the recovering of CW.

Recently, the Mexican government has promoted the modernization of this process, through the construction and innovation of the extraction of CW by applying a green process with citric acid and semi-automated equipment. In this sense, the use of this acid as an extractor agent seems to be a promising and environmentally friendly technique to replace the traditional process [1]. Since Mexico has been recognized for its potential as the main producer of CW [13], an awareness has arisen regarding the “candelilleros” (i.e., people dedicated to obtaining candelilla wax) in order to evaluate this eco-friendly alternative process. In this sense, this research was carried out in order to compare, by the first time, the chemical, structural and thermal properties of CW samples obtained from four rural communities in Mexico by two different extraction methods (i.e., traditional and eco-friendly processes).

## 2. Results and Discussion

### 2.1. Physicochemical Properties

#### 2.1.1. Basic Chemical Properties

The candelilla wax characterization allows one to evaluate the physicochemical properties and the behavior they can present in the presence of other chemical compounds. Table 1 presents the physicochemical properties of the candelilla wax from the different extraction processes. The acid value (AV) represents the natural acidity in the waxes, in other words, the sum of free fatty acids resulting from triglyceride hydrolysis. The candelilla wax refined (CWR) sample presented a lower AV (12.77 mg KOH/g), while the waxes obtained from both processes remained in a range of 16–18 mg KOH/g; similar values have been reported for raw and refined candelilla wax [14]. The AV is an indication of the quality of fats and oils, the higher the presence of free fatty acids, the higher the acidity and the lower the quality [15]. The saponification value (SV) represents the average molecular weight of the fatty acids that make up the sample. The SV remained in the range of 40–70 mg KOH/g, the lowest values were for the CWSASJ and CWSASM samples. Barbosa-Rocha et al. [16], reported similar values that obtained in this study (45–87 mg KOH/g). If the samples have short chain fatty acids, they will have a higher saponification value, because they have a higher number of carboxylic functional groups per unit mass [17]. The ester value (EV) is a measure of the amount of glyceride present in an oil sample that is saponifiable, and allows for a better approximation of the average molecular weight of triglycerides in a fat. Waxes from the CWCASJ and CWCASM regions showed lower EVs than the other samples.

The iodine number (IN) is a measure of the unsaturation present in the fatty acids that make up a triglyceride, so that if an iodine value of zero is present, the oil is fully saturated. The IN for all waxes remained in the range of 14–16 g I/100g. The IN values for the analyzed samples are into the range of 14–45 g I/100g, which were reported by other authors [16]. Although waxes are composed of long chains, they do not have many double bonds, which prevents them from being prone to oxidation. The concentration of peroxides (PV) is indicative of lipid oxidation. It is one of the main causes of off-flavor generation, spoilage and rancidity production in fats and oils. Therefore, it negatively affects their quality and shelf life [18]. The waxes from the traditional process presented higher PV values than the waxes from the eco-friendly method, due to the fact that sulfuric acid is known as an oxidant while citric acid acts as an antioxidant. However, CW from both processes showed low PV values, according to AOCS Official Method Cd 8b-90, which is applicable to all fats and oils; these values should be between 0 and 12 meq O/kg [19]. This indicates good quality CW, probably due to the presence of tannins, which act as antioxidants [20].

#### 2.1.2. Color

Table 2 shows the color parameters L*, a* and b*, where CWR and the samples from the EL and LB regions are in the light zone, with brightness values (L*) being higher than 55, except for the samples from the SJ and SM regions. In all cases, the values of a* are positive, indicating a predominance of red over green. The b* coordinate also assumes positive and relatively high values, indicating a strong predominance of yellow over blue coloration. Due to the characteristics present in candelilla wax, the Food and Drugs Administration (FDA) [21], recognizes it as safe, so it has many applications (CAS 8006-44-8). Candelilla wax is brown in its raw form, so it is more suitable to produce cosmetics, chemical products, confectionery polishes, and printer toners [20]. Once refined, the wax changes to yellow color, and can be used to develop edible coatings for fruits, food additives and clouding agents for baked goods, dressings, seeds, and candies, among others [22,23,24]. The samples from SJ and SM regions of both extraction processes presented darker waxes than the CWR and the EL and LB regions do. Thus, the obtained result suggests that the color of the wax does not depend on the extraction method, but on the characteristics of the plant and the region where it is found. In addition, the lighter waxes may not need an additional bleaching process.

### 2.2. Chemical and Structural Properties of Candelilla Wax

#### 2.2.1. Analysis by Raman Micro-Spectroscopy and Fourier Transform Infrared Spectroscopy (FTIR)

Candelilla wax consists of long-chain hydrocarbons (C29-C33) such as nonacosane, hentriacontane and tritriacontane, as well as organic esters, myricyl alcohol and organic acids in smaller proportions [14,25]. Features of these functional groups were observed by Raman and FTIR spectra.

Table 3 shows the vibrational bands of each Raman spectra of the different waxes.

The peak signal at 1063 cm^−1^ corresponding to the (C=O) group was found, followed by the peak at 1131.11 cm^−1^ (C-C), in addition to peaks at 1294.38 and 1442.90 cm^−1^ (CH_2_), which indicate the vibrational bending of methylene groups, and signals at 2844 and 2874 cm^−1^, corresponding to symmetric and asymmetric stretching of the methylene groups (CH_2_), respectively [26,27]. According to Edwards and Falk [21], the ester content is demonstrated by a weak stretching peak (C=O) around 1640 cm^−1^, which was not found in the spectra studied; however, this presence is reinforced by the peak at 1063 cm^−1^.

**Figure 1:**
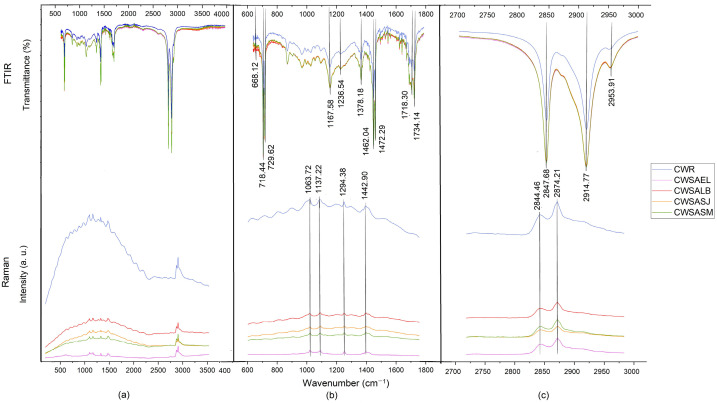
Raman and FTIR spectra of candelilla wax produced by the traditional process: (**a**) Complete; (**b**) 600–1800 cm^−1^ region; (**c**) 2700–3000 cm^−1^ regions.

**Figure 2:**
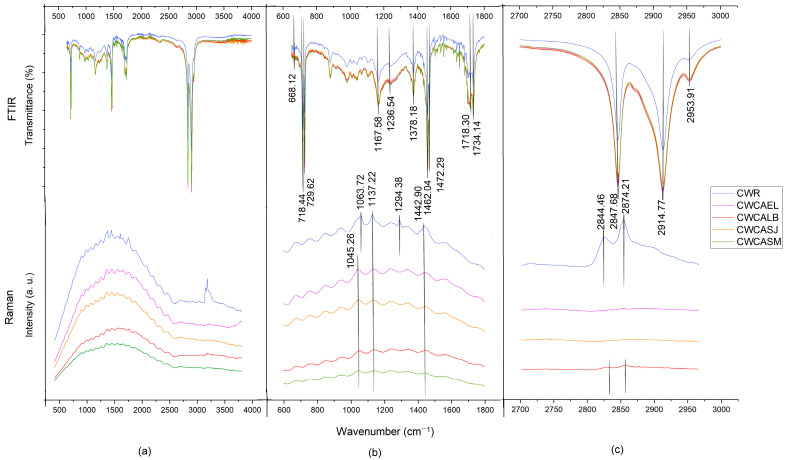
Raman and FTIR spectra of candelilla wax produced by the eco-friendly process: (**a**) Complete; (**b**) 600–1800 cm^−1^ region; (**c**) 2700–3000 cm^−1^ regions.

The Raman spectra of the waxes from the sulfuric acid extraction process showed features in common with CWR (Figure 1); in contrast, the waxes from the citric acid extraction process showed important differences, mainly in the region of 2, 700 to 3, 000 cm^−1^ (Figure 2). Edwards and Falk [21] reported the absence of the peaks at 2655 cm^−1^ (CH_3_-CH) and 1154 cm^−1^ (COH) in candelilla wax.

The lack of vibrational bands in the Raman spectra can occur due to the appearance of noise known as fluorescence, which appears as a curvature, possibly dulling the intensity of the signals. Materials that are composed of amorphous zones are more susceptible to present this phenomenon [28,29]. On the other hand, in the analysis by FTIR spectroscopy, no differences were found in the presence of functional groups between the samples and the CWR; in addition, the FTIR spectra presented more vibrational bands than the Raman spectra (Table 4).

The band 668.12 cm^−1^ indicated the bending of an aromatic ring out of plane [27], the signals 718.44 and 729.62 cm^−1^ represent the oscillating mode in the CH_2_ plane for long chain alkanes. This pair of peaks indicates crystallinity in the sample; in disordered samples, a single peak would appear at 725 cm^−1^ according to Koch and Ensikat [30]. This suggests that when the wax re-solidifies after extraction, it undergoes crystallization. The presence of long-chain alkanes is confirmed by the 1236 cm^−1^ (CH_2_) spin vibrational mode. The peak at 1167.58 cm^−1^ is related to the stretching of the C-O groups in alcohols and the double signal at 1472.29 and 1462 cm^−1^ corresponds to scissor-like vibrations in the CH_2_ groups [31]. Due to the fatty acids that make up the wax, it was expected that we would obtain signals for carbonyl groups, which were found in the range of 1734–1718 cm^−1^, corresponding to the stretching of these groups (C=O) [32]. The peak at 1378 cm^−1^ was attributed to CH_3_ symmetric deformations or OH deformation in carboxylic acid monomers; the absence of this signal in the Raman may be associated with OH signals [27]. The presence of C-H stretching bands, characteristic of saturated carbons, was present in the range of 2953–2847 cm^−1^. In recent studies, spectral signals similar to those described in this study were reported for candelilla wax [27,33]. Spectra FTIR and Raman showed notable differences in peak definition. While FTIR was able to detect the C=O vibrations associated with the esters, Raman was much more sensitive to the various CH vibrations. The waxes from both processes, presented the fluorescence effect, due to their chemical nature and the Raman effect; however, they did not present chemical differences that were associated with the extraction processes used.

#### 2.2.2. X-ray Diffraction Analysis (XRD)

**Figure 3:**
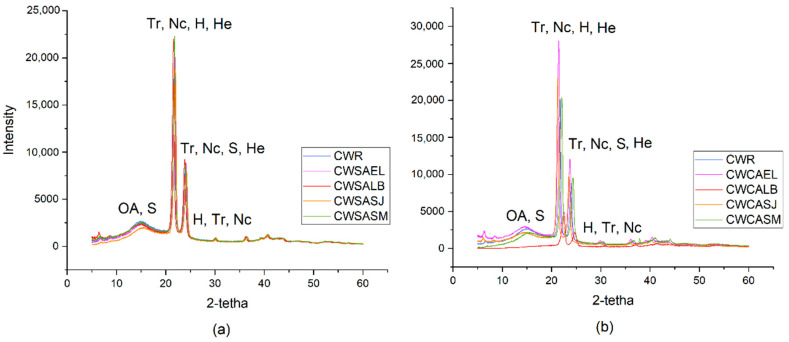
X-ray diffractograms for (**a**) Candelilla wax from the Traditional Process; (**b**) Candelilla wax from the eco-friendly Process. Tr: tritriacontane; Nc: nonacosane; H: n-heneicosane; S: sitosterol; OA: oleic acid; He: hentriacontane.

X-ray diffraction (XRD) analysis provided information on the structural characteristics of the waxes. The X-ray diffractograms of the candelilla waxes are shown in Figure 3, which present a crystalline state due to two main diffraction peaks (2θ = 21 and 24°). This pattern refers to the main signals at 4.2 and 3.8 Å in the short spacing region, which are characteristic of the orthorhombic phase presented by most epicuticular waxes [30,34]; thus, the waxes have a partially crystalline structure. The predominant constituent often defines the crystal structure of the samples; the peaks corresponding to 4.2 and 3.8 Å were found to be characteristic of odd-chain n-alkane crystals [30,35]. Due to the structural similarities of n-alkanes and the orthorhombic phase presented by candelilla waxes, this behavior was determined by its main component, n-hentriacontane (C_31_H_64_) [36].

In the diffractograms, more signals were detected, which may be related to the presence of other compounds such as oleic acid, sitosterol, nonacosane and tritracontane. As reported by Bucio et al. [33], they identified the crystalline phases of candelilla wax using the International Centre for Diffraction Data (ICDD).

The crystallinity index (CI) represents the average crystallite size, perfection, and order of a crystal. Additionally, the size of the crystal is related to the reflection emitted by a diffraction pattern from inside the unit cell of the crystal [37]. According to the calculation made for the crystal size, it was observed that the values obtained varied from 9 to 20 nm and the calculated crystallinity index presented values between 0.20 and 0.02 (Table 5).

No differences were found between the waxes of both processes, except that the CWCALB sample showed the least similarity with the CWR sample, since it obtained the smallest data in its structural properties and lower signals in the diffractograms. Several types of disorders occur in epicuticular waxes, depending on the distribution of chain length, functional groups, crystallization conditions and the concentration of the main wax components [38]. Most measurements of the crystalline order of vegetable waxes are performed on recrystallized waxes.

The size and broadening of the CWCALB diffraction peak illustrated a reduction in crystal size due to the handling of the sample, causing the development of micro-deformations. In addition, the displacement of the peak to lower values (Figure 3) indicated the presence of impurities that could interfere in the crystalline order, forming amorphous zones, which have structures that are characterized by an absence of periodicity and only the short-range order is maintained [39,40].

This behavior has already been reported [40], where they found that the long hydrocarbon chains formed a crystalline zone, while the gaps and loose ends at the edges of the crystal form an amorphous zone, due to the variation of chain lengths present. In contrast, in waxes such as kerosene wax, they have a more stable structure due to lower molecular weight components such as alkanes [41].

These structural properties demonstrated that candelilla waxes form a strong three-dimensional gelation network, and the development of microcrystalline structures, which were not affected by the extraction processes studied. The characteristics found are desirable for the formation of oleogels [42,43]

### 2.3. Thermal Properties of Candelilla Wax

Differential Scanning Calorimetry (DSC)

**Figure 4:**
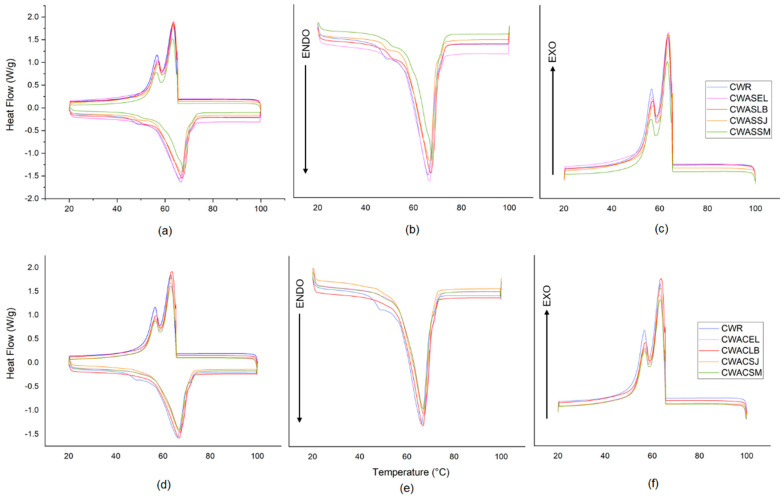
DSC thermograms of (**a**) candelilla wax from the traditional process; (**b**) heating, (**c**) cooling; (**d**) candelilla wax from the eco-friendly process; (**e**) heating, (**f**) cooling.

The complete thermograms obtained during the DCS measurements of the waxes are shown in Figure 4a,d. The cooling thermograms (Figure 4c,f) showed two exotherms that were associated with the crystallization of aliphatic acids and alcohols (higher peak) and with the crystallization of n-hentriacontane (lower peak), as reported by Serrato-Palacios et al. [44]. In contrast, the melting temperature (Tm) and the enthalpy of fusion (ΔHm), are parameters that are directly associated with the structural order of the molecules that form the three-dimensional crystalline structure of waxes [45]. In the heating thermograms (Figure 4b,e), an endotherm with an average Tm for all waxes of 66 °C and an enthalpy of fusion ∆Hm in the range of 120–152 J/g was observed (Table 6).

The Tm y ∆Hm analyzed in this study obtained values similar to those reported for candelilla wax, 66.41 °C y 147.8 J/g [44], 68.5 °C y 172.6 J/g [46], 64 °C y 144 J/g [47]. Although the Tm of CW is that reported for hentriacontane (majority component) at 99.5% purity (67.56 °C), the ∆Hm is lower (119.9 J/g) than that of CW [44,45]. It is important to remember that candelilla wax is also composed of nonacosane and tritriacontane, as observed in the XRD analysis. According to Toro-Vazquez et al. [48], these compounds could develop mixed molecular packing with hentriacontane during cooling, resulting in the two exothermic peaks present in the thermograms (Figure 4c,f) and the lower ∆Hm value for pure hentriacontane.

All waxes were subjected to a heating–cooling profile for four cycles to determine their thermal history. It was observed that in all cases, the waxes showed the same behavior for each cycle, which indicated that they are stable to temperature changes. With these properties, candelilla waxes can be used in different processes such as in the production of oleogels in combination with different oils, where it is important that the thermodynamic stability is not affected [49,50] as well as in the cosmetics industry, where they are used as an emollient and film-forming agent, improving product stability against temperature increases and extending the shelf life without any chemical change [20].

### 2.4. Mechanical Properties of Candelilla Wax

Hardness and Brittleness Measurements

Table 7 shows the mechanical properties for candelilla wax samples. The waxes from the citric acid extraction process showed hardness values above 140 N; on the other hand, the waxes from the sulfuric acid process and the CWR sample obtained values between 92 and 134 N. Candelilla wax is hard, due to its chemical structure and molecular organization, and is formed by esterified compounds, alcohols, and long-chain fatty acids [20,41]. Wax brittleness represents the force required to cause a sample to deform until it breaks during compression [46]. The values showed significant differences for both processes (Table 7).

The waxes analyzed in this study showed the same behavior; however, the CWSASJ sample showed a higher value for brittleness than for hardness. This behavior is explained by the high content of polar fractions, such as esters and fatty alcohols, that are present in candelilla wax. Lim et al. [50] and Rojas et al. [51] reported the use of waxes for the elaboration of lipid matrices and oleogels, respectively, finding that carnauba wax and candelilla wax showed a higher rigidity, but lower elasticity, indicating a more fragile behavior when compared to beeswax.

Therefore, candelilla wax has different applications; it has been used to increase the hardness of some softer waxes [46,52], as well as improving the texture of oleogels [49,53].

## 3. Materials and Methods

### 3.1. Candelilla Wax Extraction Processes

**Figure 5:**
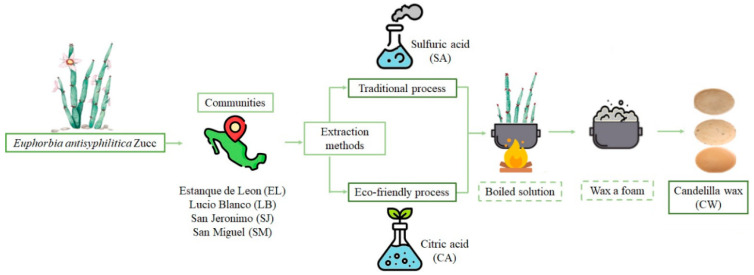
Extraction processes of candelilla wax.

This study was carried out with the collaboration of candelilla wax producers from four rural communities in 2021 (Estanque de León (26.09496° N, 102.20782° O) (EL) and Lucio Blanco, Cuatrociénegas, Coahuila (26.16924° N, 102.18839° O) (LB); San Jerónimo (24.99670° N, 102.21901° O) (SJ) and San Miguel, Melchor Ocampo, Zacatecas (24.92860° N, 102.13274° O) (SM). Samples of candelilla wax were acquired from each community, through the traditional process with sulfuric acid (SA) (0.4% initial concentration) described by Rojas-Molina [14], and the candelilla plants collected from each region were subjected to the eco-friendly process with citric acid (CA); they were obtained under the most similar conditions to the traditional process. Briefly, plant material was set into a boiling citric acid solution (2.4% initial concentration) in a steel container for approximately 40–50 min and this step was repeated four more times. In each extraction step, the wax appeared as a gray foam, which was collected and cooled. Finally, the samples were boiled twice with the correspond ding acid solution to eliminate any undesirable materials (i.e., soil and plant debris) (Figure 5). A commercially refined candelilla wax (CWR) was used as a control (Multiceras S.A de C.V. Monterrey, México).

### 3.2. Candelilla Wax Characterizations

The candelilla waxes obtained from each of the communities studied were identified by the name of the candelilla wax (CW), the extraction processes, traditional sulfuric acid (SA), and citric acid (CA), and the rural communities’ names.

#### 3.2.1. Physicochemical Properties 

##### Chemical Basic Properties

The chemical basic properties the candelilla waxes evaluated were the acid, saponification, ester, peroxide values, and iodine absorption number, and they were determined according to the American Oil Chemistry Society Method (AOCS Cd 3d-63, AOCS Cd 8b-90, AOCS Cd 3-25 and AOCS Cd 1d-92 [19,54,55,56], respectively). The ester value was determined by subtracting the acid value from the saponification value.

##### Color

Circular samples were prepared with a diameter of 5.3 cm, a height of 1.2 cm and a weight of 25 g. The color was determined using a Hunter Colorimeter fitted with an optical sensor (HunterLab ColourFlex EZ spectrophotometer), using the L*, a*, b* color system. L* is the lightness component that measures black (0) to white (100), the a* parameter goes from green to red, the b* parameter from blue to yellow, and the calibration of the equipment was carried out before use, through performing a reading on a white tile pattern (L* = 97.63, a* = 0.78 y b* = 0.25) [57]. 

### 3.3. Chemical and Structural Properties of Candelilla Wax

#### 3.3.1. Analysis of Raman Micro-Spectroscopy and Fourier Transform Infrared Spectroscopy (FTIR)

Raman spectra were recorded directly with an RMS system (Xplora Plus, Horiba, Jobin Yvon, L., Longjumeau, Cedex, France). The equipment was set up to obtain the spectra, using a 532 nm laser with a maximum power of 50 mW, 10× visible objective (MPlan N, 10×/0.25/-/FN22, Olympus), confocal coupling optics between the microscope and spectrometer, a grating of 600 g/mm, and the RMS being controlled using LabSpec6 software (Horiba, Longjumeau, Cedex, France). From each sample, two spectra were recorded, with 5 acquisitions, 30 s, in a Raman shift range of 500 to 3500 cm^−1^ [26].

The characterization of functional groups was analyzed by FTIR using an Agilent Cary 630 FTIR coupled to a zinc selenide crystal (ZnSe) ATR. The reading was performed using the MicroLab PC program in the spectral range of 4000 to 650 cm^−1^, with a cycle of 32 scans with a resolution of 2 cm^−1^ [34].

#### 3.3.2. X-ray Diffraction Analysis (XRD)

The candelilla waxes were kept at 25 °C for 24 h before analysis. The crystalline nature of all waxes was evaluated in a Miniflex 600 X-ray diffractometer (RIGAKU, Tokyo, Japan) with Ni-filtered Cu Kα radiation (λ = 1.54056 Å, 40 kV and 15 mA). The measurements were obtained with steps pf 0.02° in 2θ and every scan was recorded in the range of 5 to 90° (2θ degrees) [58]. The crystallinity index (CI) was calculated by the following formula reported by Yue-Sa et al. [37].

$$\mathrm{CI}=\left(\frac{0.24}{\beta 002}\right)$$

where β002 represents the full width at half maximum (FWHM) of (002) reflections.

Likewise, the crystallite size (CS) was obtained by Scherrer’s formula:

$$\mathrm{CS}=\left(K*\lambda \right)/\left(\beta_{½}*\theta \right)$$

where K is the crystal shape constant (0.94), λ is the wavelength of the X-ray radiation, which is 0.1542 nm (wavelength of the Cu Kα), β_1/2_ is the full width at half maximum (FWHM = 2θ degree), and θ is the Bragg angle of the plane (020).

The Raman, FTIR and XRD spectra were carried out using the OriginPro^®^ 8 program (OriginLab, MA, USA).

### 3.4. Thermal Properties of Candelilla Wax

#### Differential Scanning Calorimetry (DSC)

The thermal analysis of candelilla waxes was performed using a Q2000 differential scanning calorimeter (TA Instruments, New Castle, DE, USA). Samples (3–5 mg) were placed into an aluminum hermetic pan. For the crystallization and melting profiles, the samples were first heated over the range of 20 to 100 °C at 5 °C/min, and then cooled over the range of 100 to 20 °C at 5 °C/min, to remove their thermal history [59]. Each sample was subjected to four cycles of heating and cooling under the same conditions. Thermal properties, including the melting temperature (Tm), melting enthalpy (ΔHm), crystallization temperature (Tc), and crystallization enthalpy (ΔHc), were computed from thermograms using Universal Analysis 2000 software. The reported data correspond to the average and standard deviation (SD) of two replicates for each sample.

### 3.5. Mechanical Properties of Candelilla Wax

#### Hardness and Brittleness Measurements

A Texture Analyzer TA-XT Plus was used to determine the hardness and brittleness values of the waxes. All samples were placed into the sample holders and the probe was positioned over the center of the cylindrical samples (5.5 cm diameter × 0.5 cm height), placed horizontally, and moved down some millimeters at 1 mm/s. Samples were evaluated three times for each sample at room temperature (25 °C). Hardness was measured by a penetration test with a Warner Bratzler cylinder probe (10 cm length; 280.97 g); the brittleness test was conducted with a plate probe (15 cm length; 77.5 g) [60].

## 4. Conclusions

The production of candelilla wax is one of the most important economic activities in Northern Mexico. However, the conventional extraction method uses sulfuric acid, which results in environmental concerns, represents risks to health and reduces the quality of the product, since it still needs to be filtered to eliminate residues and subjected to a bleaching process for its commercialization. Therefore, an alternative technology was proposed, in which the corrosive extracting agent is replaced by citric acid that does not emit toxic gases.

The physicochemical properties of the waxes obtained by both processes were analyzed in four producing communities in Mexico. Candelilla wax from the eco-friendly process showed significant differences in its physicochemical properties and improvements in mechanical properties when compared to candelilla wax from the traditional process. Therefore, the green process can be considered viable, since it increased the quality of the waxes obtained under the extraction conditions experimented, so that producers can save the steps of the elimination of impurities by filtration and the bleaching process for extensive industrial use.

## Figures and Tables

**Table 1:** Basic chemical properties of candelilla wax extracted by two different methods.

Regions	Candelilla Wax	Physicochemical Properties Values				
		Acid	Saponification	Ester	Iodine Absorption Number	Peroxide
		(mg KOH/g)	(mg KOH/g)	(mg KOH/g)	(g L/100 g)	(meq O/kg)
Estanque de León	CWSAEL	17.32 ± 0.23	71.03 ± 10.7	53.72 ± 10.87	14.14 ± 0.14	0.406 ± 0.099
	CWCAEL	17.75 ± 0.56	62.16 ± 11.53	44.41 ± 11.36	15.38 ± 0.26	0.076 ± 0.030
Lucio Blanco	CWSALB	16.82 ± 0.33	58.83 ± 6.67	42.01 ± 6.67	15.58 ± 1.46	0.225 ± 0.011
	CWCALB	18.49 ± 0.27	69.93 ± 1.94	51.45 ± 1.82	16.26 ± 0.65	0.126 ± 0.013
San Jerónimo	CWSASJ	17.45 ± 0.15	40.41 ± 1.66	22.96 ± 1.77	15.34 ± 0.52	0.346 ± 0.002
	CWCASJ	17.38 ± 0.14	61.05±2.78	43.68 ± 2.84	15.08 ± 1.07	0.091 ± 0.046
San Miguel	CWSASM	17.11 ± 0.50	39.95 ± 7.7	22.83 ± 8.18	14.31 ± 0.24	0.209 ± 0.080
	CWCASM	17.85 ± 0.63	62.83 ± 1.17	44.97 ± 1.53	14.65 ± 0.22	0.131 ± 0.030
	CWR	12.77 ± 0.47	67.70 ± 6.92	54.92 ± 7.30	13.91 ± 0.88	0.139 ± 0.003
Reference values		12–24 [14]	45–87 [16]		14–45 [16]	0–12 [19]

CW: candelilla wax; SA: sulfuric acid; CA: citric acid; EL: Estanque de León; LB: Lucio Blanco; SJ: San Jerónimo; SM: San Miguel; CWR: Candelilla wax refined.

**Table 2:** Color parameters of candelilla wax produced by different process.

Candelilla Wax	Hunter			
	L*	a*	b*	Color
CWSAEL	63.95	3.2	26.46	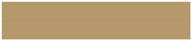
CWSALB	58.27	8.05	34.27	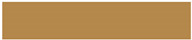
CWSASJ	55.99	5.64	29.61	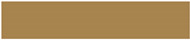
CWSASM	51.07	10.08	33.48	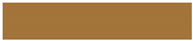
CWCAEL	70.21	3.02	24.34	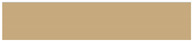
CWCALB	75.88	2.52	25.40	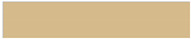
CWCASJ	55.38	5.81	27.57	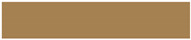
CWCASM	47.20	8.79	31.20	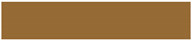
CWR	62.06	10.53	37.95	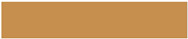

CW: candelilla wax; SA: sulfuric acid; CA: citric acid; EL: Estanque de León; LB: Lucio Blanco; SJ: San Jerónimo; SM: San Miguel; CWR: Candelilla wax refined.

**Table 3:** Raman spectral bands and molecular assignment in candelilla waxes.

Regions	Candelilla Wax	Approximate Assignment of Vibrational Mode (Wavenumber cm^−1^)					
		CH_2_ Asymmetric	CH_2_ Symmetric	CH_2_	CH_2_ Twisting	C-C	C=O
Estanque de León	CWSAEL	2874.21	2844.46	1442.90	1294.38	1137.22	1063.72
	CWCAEL	NF	NF	1442.90	NF	1137.22	1045.26
Lucio Blanco	CWSALB	2874.21	2844.46	1442.90	1294.38	1137.22	1063.72
	CWCALB	2874.21	2844.46	1442.90	NF	1137.22	1045.26
San Jerónimo	CWSASJ	2874.21	2844.46	1442.90	1294.38	1137.22	1063.72
	CWCASJ	NF	NF	1442.90	NF	1137.22	1045.26
San Miguel	CWSASM	2874.21	2844.46	1442.90	1294.38	1137.22	1063.72
	CWCASM	NF	NF	1442.90	NF	1137.22	1045.26
	CWR	2874.21	2844.46	1442.90	1294.38	1137.22	1063.72

CW: candelilla wax; SA: sulfuric acid; CA: citric acid; EL: Estanque de León; LB: Lucio Blanco; SJ: San Jerónimo; SM: San Miguel; CWR: Candelilla wax refined.

**Table 4:** FTIR bands and their vibrational assignment from all candelilla waxes.

	Approximate Assignment of Vibrational Mode	
Wavenumber (cm^−1^)	Group	Assignment
2953.91	CH_3_	stretching
2914.77	CH_3_	stretching
2847.68	CH_2_	stretching
1734.14	C=O	stretching
1718.30	C=O	stretching
1472.29	CH_2_	scissoring
1462.04	CH_2_	bending
1378.18	CH_3_	symmetric deformation
1236.54	CH_2_	twisting
1167.58	C-O	bending
729.62	CH_2_	In-plane rocking
718.44	CH_2_	In-plane rocking
668.12	Aromatic ring	Out of plane ring bending

**Table 5:** X-ray diffraction properties of candelilla waxes.

Regions	Candelilla Wax	FWHM		Crystallinity Index (CI)		Crystal Size (nm)	
Estanque de León	CWSAEL	0.48	0.57	0.12	0.07	17.58	14.81
	CWCAEL	0.43	0.52	0.17	0.09	19.65	16.25
Lucio Blanco	CWSALB	0.45	0.54	0.14	0.08	18.69	15.45
	CWCALB	0.71	0.86	0.03	0.02	11.79	9.84
San Jerónimo	CWSASJ	0.51	0.60	0.10	0.06	16.55	14.10
	CWCASJ	0.40	0.50	0.20	0.11	20.64	16.96
San Miguel	CWSASM	0.48	0.57	0.14	0.07	18.34	15.10
	CWCASM	0.51	0.59	0.10	0.06	16.52	14.20
	CWR	0.46	0.56	0.13	0.07	18	14.99

FWHM: represents the full width at half maximum of (002) reflections. CW: candelilla wax; SA: sulfuric acid; CA: citric acid; EL: Estanque de León; LB: Lucio Blanco; SJ: San Jerónimo; SM: San Miguel; CWR: Candelilla wax refined.

**Table 6:** Thermal parameters obtained from the cooling and melting thermograms of candelilla wax.

Regions	Candelilla Wax	T_m_ (°C)	∆H_m_ (J/g)	T_c_ (°C)	∆H_c_ (J/g)
Estanque de León	CWSAEL	66.11 ± 0.01	137.32 ± 0.12	63.54 ± 0.04	128.2 ± 0.12
	CWCAEL	66.84 ± 0.02	141.8 ± 0.18	63.29 ± 0.01	131.45 ± 0.11
Lucio Blanco	CWSALB	66.38 ± 0.05	142.85 ± 0.23	63.44 ± 0.02	136.6 ± 0.14
	CWCALB	66.42 ± 0.02	146.52 ± 0.05	63.72 ± 0.01	137.07 ± 0.12
San Jerónimo	CWSASJ	66.41 ± 0.29	105.22 ± 0.05	63.72 ± 0.03	136.32 ± 0.20
	CWCASJ	66.37 ± 0.14	142.75 ± 2.82	63.47 ± 0.04	134.55 ± 0.15
San Miguel	CWSASM	66.39 ± 0.06	119.3 ± 0.14	63.21 ± 0.02	111.85 ± 0.05
	CWCASM	66.48 ± 0.03	132.3 ± 0.14	63.33 ± 0.03	123.65 ± 0.15
	CWR	65.79 ± 0.01	151.57 ± 0.15	63.25 ± 0.05	141.52 ± 0.12

T_m_: Melting temperature; ∆H_m_: Melting enthalpy; T_c_: Crystallization temperature; ∆H_c_: Crystallization enthalpy. CW: candelilla wax; SA: sulfuric acid; CA: citric acid; EL: Estanque de León; LB: Lucio Blanco; SJ: San Jerónimo; SM: San Miguel; CWR: Candelilla wax refined. Values represent the mean and standard deviation of four replicates.

**Table 7:** Mechanics properties of candelilla wax produced by different process.

Candelilla Wax	Hardness (N)	Brittleness (N)
CWSAEL	134.74 ± 11.27	42.09 ± 28.97
CWSALB	131.8 ± 6.32	29.46 ± 2.22
CWSASJ	105.82 ± 10.11	113.47 ± 18.64
CWSASM	92.52 ± 3.89	36.02 ± 2.59
CWCAEL	141.72 ± 13.42	39.86 ± 12.92
CWCALB	153.18 ± 2.94	71.39 ± 23.36
CWCASJ	138.32 ± 8.94	84.22 ± 14.81
CWCASM	166.06 ± 3.12	27.35 ± 6.26
CWR	123.57 ± 17.46	69.61 ± 12.63

CW: candelilla wax; SA: sulfuric acid; CA: citric acid; EL: Estanque de León; LB: Lucio Blanco; SJ: San Jerónimo; SM: San Miguel; CWR: Candelilla wax refined. Values represent the mean and standard deviation of two replicates.

## Data Availability

Not applicable.

## Sample Availability

Not available.
